# The effects of Medicaid expansion on job loss induced mental distress during the COVID-19 pandemic in the US

**DOI:** 10.1016/j.ssmph.2022.101279

**Published:** 2022-10-29

**Authors:** Sankar Mukhopadhyay

**Affiliations:** a Department of Economics (MS – 030), University of Nevada, Reno, NV, 89557, USA; b IZA, Bonn, Germany

**Keywords:** Job loss, Depression, Anxiety, Mental health, COVID-19, Medicaid

## Abstract

The COVID-19 pandemic led to an unprecedented level of job losses in the U.S., where job loss is also associated with the loss of health insurance. This paper uses data from the 2020 Household Pulse Survey (HPS) and difference-in-difference (DD) regressions to estimate the effect of Medicaid expansion on anxiety and depression associated with job loss. Estimates show that the respondents who live in expansion states are 96.6% *more* likely to have Medicaid coverage, and 14.2% *less* likely to have moderate to severe mental distress following their job loss compared to those living in non-expansion states. The corresponding numbers associated with a family member's job loss are 36.3% and 7.6%, respectively. Next, we explore the mechanisms which suggest that the economic security provided by Medicaid is as important (if not more) as the access to or utilization of healthcare. The difference-in-difference-in-difference (DDD) estimates using just above and below the Medicare eligibility age (65) confirm these results.

## Highlights

• This paper estimates the effect of Medicaid expansion on mental health.
• We use the Household Pulse Survey and difference-in-difference regressions.
• Respondents in expansion states are 14.2% *less* likely to have mental distress following their job loss.
• The difference-in-difference-in-difference estimates confirm these results.
• Economic security provided by Medicaid is as important as access to healthcare.

## 1 Introduction

The COVID-19 pandemic led to unprecedented job destruction and a historic increase in mental distress around the world. Symptoms of anxiety or depression almost quadrupled (from 11% to 42%) between 2019 and 2020 (Abbott, 2021) in the U.S. One of the contributing factors may be the unprecedented level of job loss during the pandemic. The number of new weekly unemployment claims peaked at around 6.5 million in the first week of April 2020, and more than 36 million American workers filed for unemployment insurance in just the first eight weeks of the pandemic.^1^ The unemployment rate in the U.S. peaked at 14.8% in April 2020 and was at 6.1% in April 2, 021.^2^

Job losses can be traumatic for individuals and families. A recent survey indicates that 70% of unemployed workers report being more stressed than usual, and 56% reported having more mental health issues than usual.^3^ Research has shown that job losses not only adversely affect economic and social outcomes (Charles & Stephens, 2004) but also hurt physical and mental health (Winkelmann & Winkelmann, 1998; Cygan-Rehm et al., 2017; Clark et al., 2001; Schaller & Stevens, 2015) and even mortality (Eliason & Storrie, 2009; Sullivan & Von Wachter, 2009; Browning & Heinesen, 2012; Bloemen Hochguertel & Zweerink, 2018). In a recent paper, Johnston, Shields, & Suziedelyte (2020) found that even job insecurity has a significant negative impact on the mental health of employees.

In the U.S., job loss can be particularly stressful because it is also associated with the loss of health insurance. In the U.S., most nonelderly adults are covered by Employer-Sponsored Insurance (ESI). Estimates suggest that 7.7 million workers (and 6.9 million dependents) lost ESI in just the first three months of the pandemic (Fronstin & Woodbury, 2020). Loss of health insurance can be stressful at any time, but it can be particularly stressful in the middle of a pandemic.

Before the Affordable Care Act (ACA), those who lost ESI had limited options to acquire health insurance. However, after the ACA, those who lose ESI may be eligible for a subsidy to buy insurance from the Health Insurance Exchanges (HIX) in all states. Furthermore, they may enroll in Medicaid, especially if they live in a state that expanded the Medicaid program (36 states and Washington DC as of 2020)^4^ and their income is below 138 percent of the federal poverty limit (FPL). Recent evidence suggests that the ACA has reduced the likelihood of being uninsured by six percentage points after losing a job (Agarwal & Sommers, 2020). About 60% of the reduction is due to the expansion of Medicaid, and the rest is driven by HIX (Agarwal & Sommers, 2020).

This paper uses data from the 2020 Household Pulse Survey (HPS), a nationally representative rapid response survey, and a difference-in-difference (DD) structure to show that Medicaid expansion mitigated job loss's adverse effects on mental health during this pandemic. We use the first 27 rounds of the HPS covering the period from April 2020 to March 2021. We do not use data after March 2021 because the American Rescue Plan Act (ARPA) was passed in March 2021. The ARPA changed both Medicaid and HIX subsidies in important ways; therefore, combining the before-ARPA and after-ARPA periods is problematic.

The HPS administered the Patient Health Questionnaire-4 (PHQ-4) to assess respondents' mental health status. We use responses to these questions to compare the mental health outcomes of individuals who experienced a job loss (either their job or that of a family member) during the COVID-19 pandemic to those who did not in the expansion and non-expansion states. Previous research has shown that access to Medicaid can improve mental health outcomes (Baicker et al., 2013) in the general population, but this paper aims to explore whether Medicaid mitigated a job loss's mental health consequences during the COVID-19 pandemic and the mechanisms behind it. This is an important question because one of the goals of social safety net programs is to work as a shock absorber in times of crisis. Therefore, how the ACA in general, and Medicaid expansion in particular, fared in that respect is an important question.

Next, we explore mechanisms. Medicaid may mitigate the adverse effects of job loss on mental health through two primary mechanisms. First, individuals may be able to access/utilize mental health services and prescription medications, reducing mental distress. An alternative explanation is that lower costs (and expected costs) associated with Medicaid may lower financial stress and food insecurity. Previous research has also shown that financial hardship (Butterworth et al., 2009) and especially food insecurity (Siefert et al., 2004; Weaver et al., 2021; Jones, 2017; Lund et al., 2010; Tribble et al., 2020) is associated with poor mental health. The relationship between food insecurity and poor mental health outcomes persists even after controlling for socioeconomic status (Elgar et al., 2021; Sorsdahl et al., 2011). The exact mechanism behind this relationship remains unclear. Weaver et al. (2021) suggest that the psychological effects of not having enough or desired food and the uncertainty associated with future meals are more likely to explain the relationship between food insecurity and poor mental health than a nutritional deficiency. Therefore, we explore mechanisms through which Medicaid expansion affects the mental health of Americans.

We present our results in two steps. First, we show that individuals in expansion states are 14.2% (7.6%) less likely to have moderate to severe mental distress following their job loss (job loss of a family member) than those living in non-expansion states. Next, we show that individuals in expansion states are more likely to be covered by Medicaid following a job loss. Our estimates show that the respondents who live in expansion states are 96.6% (36.3%) more likely to have Medicaid coverage following their (family member's) job loss.

Further analysis shows that individuals living in expansion states are about 9.2% less likely to be in financial distress and 13.6% less likely to be food insecure than their counterparts in non-expansion states. Therefore, economic security is a critical mechanism through which Medicaid reduces mental distress. We find limited evidence in favor of increased healthcare utilization in expansion states.

One potential problem with the above approach is that there may be differences in access to other social safety programs across states that affect unemployed people in expansion and non-expansion states differently. Then the DD would be biased. To account for that, we compare the effect of Medicaid expansion on respondents just below the age of 65 (63–64) and just above 65 (66–67) and estimate a Difference-in-Difference-in-Difference (DDD) model. While we expect Medicaid expansion to affect 63-64-year-old respondents, there will be little or no effect among 66-67-year-old individuals since they are eligible for Medicare. Furthermore, most social safety programs (such as worker protection or food assistance) are not age-restricted. In addition, these individuals are of similar age, and therefore, we would expect their financial and health profiles to be similar. Now, any difference in state-level variation in safety programs will be differenced out. We show that our DDD estimates are similar to our DD estimates.

The rest of the paper is structured as follows. Section 2 explains some background materials, Section 3 describes the data, Section 4 discusses the results, and Section 5 concludes.

## 2 Background

The ACA created HIX in all states, but only some states (36 states and Washington DC as of 2020) expanded Medicaid coverage. Therefore, the central premise of this paper depends on Medicaid being more beneficial for mental health than health insurance brought through HIX. There may be several reasons; some are general, and some are specific to the COVID-19 pandemic.

First, Medicaid eligibility is based on current income. Therefore, even individuals with a relatively high income in 2019 may become eligible for Medicaid if they lose their job and their income falls below the cutoff level since the ACA removed the asset test for Medicaid eligibility. On the other hand, the HIX subsidy is determined by annual income. Therefore, even a few months of high income (for example, in the first three months of 2020, before COVID-19 became a pandemic in the U.S.) may substantially reduce the subsidy.

Second, the Coronavirus Aid, Relief, and Economic Security (CARES) Act of 2020 stipulated that the COVID-19-specific additional unemployment benefits ($600 per week until July 2020, $300 per week as of March 2021) do not count as income to determine Medicaid eligibility, but that benefit counts as income to determine HIX subsidy.^5^ This puts HIX consumers at a disadvantage compared to the Medicaid population.

Furthermore, Medicaid has very little or no cost-sharing requirements (Brooks et al., 2019) in most states, which may mean that the out-of-pocket cost of Medicaid beneficiaries may be lower than those with HIX plans, even after accounting for the COVID-19-related expansion of Annual Premium Tax Credit (APTC) and Cost Sharing Reduction (CSR) subsidies associated with HIX plans. These Out-of-pocket costs are a big concern, even for individuals and families with health insurance. More than 40% of all nonelderly adults in the U.S. report financial distress induced by medical expenditure (Doty et al., 2008). A majority (61%) of these individuals experienced medical expenditure induced financial hardship despite having health insurance. This suggests that just having health insurance is not enough. It has to be affordable.

Moreover, 29% of all nonelderly adults reported not paying for basic needs such as food or rent because medical expenditure induced financial hardship (Doty et al., 2008). Over the past few years, research has shown that Medicaid expansion has reduced out-of-pocket expenditures (Golberstein et al., 2015), reduced unpaid medical bills (Hu et al., 2016), improved credit scores, and reduced bankruptcy (Miller, Hu, Kaestner, Mazumder, & Wong, 2021) have reported that Medicaid expansion may reduce food insecurity (Himmelstein, 2019).

Third, The CARES Act increased the federal medical assistance percentage (FMAP) by 6.2 percentage points. However, this increase did not apply to the population covered under the ACA Medicaid expansion. Therefore, this would have affected expansion and non-expansion states symmetrically. However, the CARES Act of 2020 also stipulated that to be eligible for the 6.2 percentage point increase in FMAP, states must provide continuous eligibility to all Medicaid recipients who become eligible for Medicaid on or after March 18, 2020, as long as the COVID-19 public health emergency (PHE) is in effect. The PHE was in effect for our entire sample period. This continuous eligibility meant that once in Medicaid, individuals could remain in Medicaid irrespective of any changes in their circumstances. Please note that while the increased FMAP did not apply to the ACA population, the continuous coverage mandate did.

## 3 Data and empirical strategy

### 3.1 Data

We use data from the 2020 Household Pulse Survey (HPS); a nationally representative rapid response survey conducted by the U.S. Census Bureau and designed to measure the effects of COVID-19 in the U.S. It was conducted weekly from April to July 2020 and then bi-weekly. We use 27 rounds of the HPS covering the period from April 2020 to March 2021. As we mentioned before, we do not use data after March 2021 because the American Rescue Plan Act (ARPA) was passed in March 2021.

The HPS contains information on the respondents' mental health and a plethora of demographic and socioeconomic information on responding households. Our primary focus is on nonelderly adults (age<65) since individuals above 65 are eligible for Medicare and are unlikely to be affected by Medicaid expansion.

Our data consists of 1,724,069 person-round observations. Among them, 1,507,313 respondents have valid (non-missing) observations for all four questions about mental distress (PHQ-4). We exclude individuals who are voluntarily not working, retired, disabled, or lost jobs before the COVID-19 pandemic (207,811 observations). We do so by using three relevant questions in the HPS. The first question asked, “*Have you, or has anyone in your household experienced a loss of employment income since March 13, 2020?”* The second question asked, *“Now we are going to ask about your employment. In the last 7 days, did you do ANY work for either pay or profit?”* If a respondent answered “Yes” to the first question and “No” to the second question, then they are in the “own-job-loss” group (G1). If a respondent answered “Yes” to the first question and “Yes” to the second question, then they are in the “family-member-job-loss” group (G2). These two groups are more likely to be affected by Medicaid expansion. If an individual and a family member lost their jobs, they are in G1. We cannot separate them from the “own-job-loss” group (G1), given that questions about the labor force participation status of the family members were not asked in the HPS. If a respondent answered “No” to the first question and “Yes” to the second question, then neither they nor their family members lost a job (third group or G3). This group is unlikely to be affected by Medicaid expansion since they are (most likely) covered by Employer-Sponsored Insurance (ESI). Therefore, comparing the mental distress of G1 (or G2) with G3 in expansion and non-expansion states may allow us to estimate the effect of Medicaid expansion on job-loss-induced mental distress.

If respondents answered “No” to both questions, they are dropped from our sample because the job loss happened before COVID-19. We exclude individuals who lost jobs before the COVID-19 pandemic because these job losses may be tied to individual-specific causes. On the other hand, most job losses during the COVID-19 pandemic were mass layoffs (Dias et al., 2020; Hernández, 2020). The individuals who reported not working (i.e., answered “No” to the second question) were asked about the reason for not working. The exact question was, *“What is your main reason for not working for pay or profit?”* If the answer was “did not want to be employed at this time”, “disabled”, or “retired” then they are dropped from the sample since our focus is on individuals who experienced a job loss.

Finally, a further 427,074 observations are excluded because of missing control variables, leaving us with a final sample size of 872,428. Despite the reduction in sample size, the analysis sample remains representative (please see Table 1). We present the summary statistics of the analytical sample in Table 2.

**Table 1:** Representativeness of the analysis sample.

	Analysis sample	Maximum possible sample size
Age	43.92	44.92
	(11.25)	(11.88)
Female	0.60	0.61
	(0.49)	(0.49)
Married	0.57	0.57
	(0.49)	(0.49)
# Children	0.85	0.83
	(1.14)	(1.14)
Sample size	872,428	1,724,069

Hispanic	0.09	0.10
	(0.28)	(0.31)
Sample size	872,428	1,724,069
	
Race		
White	0.81	0.81
	(0.39)	(0.40)
Black	0.08	0.09
	(0.28)	(0.29)
Asian	0.05	0.05
	(0.24)	(0.23)
Other	0.06	0.05
	(0.23)	(0.23)
Sample size	872,428	1,683,470

Education		
Less than HS	0.004	0.007
	(0.07)	(0.08)
Some HS	0.01	0.02
	(0.10)	(0.13)
HS degree	0.10	0.12
	(0.30)	(0.32)
Some college	0.20	0.21
	(0.40)	(0.41)
Assoc. degree	0.11	0.11
	(0.31)	(0.31)
College deg.	0.32	0.30
	(0.47)	(0.46)
Graduate deg.	0.25	0.24
	(0.43)	(0.42)
Sample size	872,428	1,718,685

**Table 2:** Summary statistics: by group and by expansion status.

	Lost their jobs		A family member lost their job		No loss of jobs	
	Expansion	Not	Expansion	Not	Expansion	Not
Age	44.00	43.93	43.94	43.93	43.78	44.16
	(11.88)	(11.81)	(11.31)	(11.21)	(10.95)	(11.13)
Female	0.65	0.66	0.60	0.61	0.58	0.58
	(0.477)	(0.474)	(0.490)	(0.488)	(0.494)	(0.493)
Married	0.47	0.48	0.59	0.60	0.60	0.61
	(0.499)	(0.500)	(0.491)	(0.490)	(0.491)	(0.489)
# Children	0.88	0.95	0.85	0.90	0.82	0.83
	(1.192)	(1.224)	(1.134)	(1.152)	(1.109)	(1.111)
Race						
White	0.75	0.72	0.82	0.79	0.83	0.82
	(0.431)	(0.450)	(0.384)	(0.406)	(0.372)	(0.384)
Black	0.10	0.19	0.06	0.12	0.06	0.10
	(0.300)	(0.390)	(0.246)	(0.329)	(0.232)	(0.305)
Asian	0.06	0.03	0.05	0.03	0.06	0.04
	(0.242)	(0.170)	(0.226)	(0.171)	(0.245)	(0.187)
Other	0.08	0.06	0.06	0.05	0.05	0.04
	(0.276)	(0.246)	(0.240)	(0.227)	(0.207)	(0.196)
Education						
Less than HS	0.01	0.01	0.00	0.01	0.00	0.00
	(0.100)	(0.100)	(0.0647)	(0.0741)	(0.0455)	(0.0513)
Some HS	0.02	0.03	0.01	0.01	0.01	0.01
	(0.155)	(0.169)	(0.102)	(0.117)	(0.0733)	(0.0857)
HS degree	0.16	0.17	0.10	0.12	0.07	0.09
	(0.366)	(0.379)	(0.305)	(0.320)	(0.261)	(0.280)
Some college	0.29	0.29	0.22	0.24	0.16	0.18
	(0.452)	(0.454)	(0.415)	(0.426)	(0.363)	(0.385)
Assoc. degree	0.12	0.14	0.11	0.13	0.09	0.10
	(0.329)	(0.345)	(0.319)	(0.331)	(0.284)	(0.303)
College deg.	0.27	0.24	0.31	0.29	0.35	0.34
	(0.442)	(0.427)	(0.463)	(0.455)	(0.475)	(0.474)
Graduate deg.	0.13	0.12	0.24	0.21	0.33	0.28
	(0.338)	(0.324)	(0.424)	(0.407)	(0.470)	(0.449)
Income						
<25K	0.21	0.25	0.08	0.10	0.04	0.05
	(0.409)	(0.431)	(0.269)	(0.306)	(0.192)	(0.228)
$25K - $35K	0.13	0.14	0.08	0.10	0.05	0.07
	(0.338)	(0.350)	(0.274)	(0.303)	(0.217)	(0.248)
$35K - $50K	0.13	0.14	0.11	0.12	0.08	0.10
	(0.342)	(0.349)	(0.311)	(0.329)	(0.268)	(0.298)
$50K - $75K	0.17	0.17	0.18	0.19	0.15	0.18
	(0.380)	(0.380)	(0.384)	(0.395)	(0.361)	(0.385)
$75K - $100K	0.12	0.11	0.16	0.16	0.15	0.16
	(0.330)	(0.316)	(0.364)	(0.362)	(0.360)	(0.368)
$100K - $150K	0.13	0.11	0.20	0.18	0.23	0.22
	(0.332)	(0.307)	(0.402)	(0.382)	(0.422)	(0.414)
$150K - $200K	0.05	0.04	0.10	0.07	0.13	0.11
	(0.218)	(0.198)	(0.295)	(0.259)	(0.335)	(0.307)
>$200,000K	0.05	0.03	0.10	0.07	0.17	0.11
	(0.209)	(0.184)	(0.295)	(0.259)	(0.372)	(0.318)
Hispanic	−0.11	−0.13	−0.09	−0.11	−0.06	−0.08
	(0.317)	(0.337)	(0.283)	(0.312)	(0.245)	(0.265)
Daily # of cases	146.01	181.14	163.81	201.48	159.43	192.46
	(189.5)	(179.5)	(210.8)	(203.7)	(203.6)	(199.8)
Daily # of deaths	3.18	2.58	2.92	2.72	3.02	2.64
	(3.941)	(2.036)	(3.540)	(2.353)	(3.685)	(2.384)
*N*	115369	43750	195536	73187	323078	121508

Note: S.D. in parentheses.

Several studies on the effects of the Medicaid expansion (using the expansion of Medicaid under the ACA) on various outcomes focus on individuals with high school or less education because they are more likely to be eligible for Medicaid. However, the massive job destruction during the COVID-19 pandemic meant a wider array of people became eligible for Medicaid. For example, less than half of the respondents on Medicaid had high school (or less) education in our data. Therefore, in our baseline analysis, we included all respondents. We report estimates for respondents with high school or less education as a robustness check. The results are similar to the full sample.

The HPS administered the PHQ-4 questionnaire to assess respondents' mental health status. This questionnaire has four questions, and each is answered on a zero to three scale. The first two questions asked the respondents how anxious and worried they were, and the last two questions asked how often they felt depressed or uninterested during the two weeks before an interview. A combined score of three or more from the first two questions suggests moderate or severe anxiety, and a combined score of three or more from the last two questions suggests moderate or severe depression. Finally, a combined score of six or more from all four questions suggests moderate to severe mental distress. We use these three variables as our outcome variables to explore how Medicaid expansion mitigated the effects of job loss on mental distress.

### 3.2 Empirical strategy

Our goal is to find the effect of Medicaid expansion on job-loss-induced mental distress. Therefore, conceptually, individuals living in the expansion states have the potential to receive treatment, and individuals living in the non-expansion states are in the control group. Our framework differs from the canonical DD framework since we do not have a “before-after” structure. Not all individuals living in the expansion states receive the treatment. For expositional convenience, we are only discussing the DD structure for those individuals who lost their jobs (G1). The logic for those who did not lose their job but have a family member who lost a job during the pandemic (G2) is similar. Since the expansion states individuals who lost their jobs are affected by Medicaid expansion, they receive the treatment. Therefore, this group is comparable to the treatment group in the “after” period in the canonical DD structure. On the other hand, the expansion states individuals who did not lose jobs are not affected by Medicaid expansion. Therefore, this group is comparable to the treatment group in the “before” period in the canonical DD structure. In a similar vein, the individuals of the non-expansion states who lost (did not lose) are comparable to the control group in the “after” (“before”) period in the canonical DD structure. Therefore, in this framework, the difference between the mental health outcomes of G1 and G3 in the expansion states provides us with an estimate of the effect of job loss on mental health in the presence of access to Medicaid. The difference between the mental heal outcomes of G1 and G3 in the non-expansion states provides us with an estimate of the effect of job loss on mental health in the absence of access to Medicaid. Therefore, the difference between these two differences (known as the DD estimate) estimates the effect of Medicaid expansion on job-loss-induced mental distress.

Along with the mean DD (as described above), we also use DD regressions to estimate the effect of Medicaid expansion after controlling for several control variables and state-level fixed effects.

We estimate the following regression

(1) $$O_{i}=\delta X_{i}+S_{i}+{\beta_{1}G}_{1i}+{\beta_{2}G}_{2i}+{\gamma_{1}G}_{1i}*{Expan}_{i}+{\gamma_{2}G}_{2i}*{Expan}_{i}+\varepsilon_{i}$$

In regressions, $O_{i}$ is the outcome variable. We control for several demographic, socioeconomic variables, and round fixed effects ($X_{i}$). We also include state of residence ($S_{i}$) fixed effects. In addition to individual and household characteristics, we also control for local COVID-19 conditions using Johns Hopkins University (JHU) data on COVID-19 activity. Since the state dummies are included in the regressions, the parameters are identified from within state variations. Our primary parameters of interest are $\gamma_{1}$ and $\gamma_{2}$, which are the DD estimates of the effect of Medicaid expansion on job-loss-induced mental distress. Since most of our outcome variables are categorical, we use Logit or Ordered Logit models and report the estimated odds ratios. All standard errors are clustered at the state level.

The DD estimates are causal if the trends in the treatment and control groups would have been the same without the intervention (parallel trends assumption). Testing this assumption is never feasible. Instead, if researchers have multiple periods of pre-intervention data for both groups, they can test whether the trends in treatment and control groups had similar trends before the treatment or intervention. We do not have a before-after structure. Therefore, the traditional way of checking for parallel trends is not feasible in our context. Therefore, we rely on placebo testing. We do this in multiple ways:

1. We show that the prevalence of COVID-19 and its impact on the economy (as proxied by the rate of job loss or program participation such as the Supplemental Nutrition Assistance Program or SNAP) was similar in expansion and non-expansion states
2. We show that there is no treatment effect on variables where we would not expect any treatment effect (such as demographic characteristics such as race, gender, etc.).
3. This still leaves the possibility of unobserved state-level differences, such as the availability of other social safety net programs. If these differences affect employed and unemployed similarly, state fixed effects will account for that. However, if there are differences in other social safety programs across states that affect unemployed people in expansion and non-expansion states differently, the DD estimates may be biased. To account for that, we compare the effect of Medicaid expansion on respondents below the age of 65 (63–64) and above 65 (66–67) and estimate a Difference-in-Difference-in-Difference (DDD) model. We picked these age windows to exploit the fact that an individual becomes eligible for Social Security at age 62, though at a reduced benefit. For example, an individual born in 1958 would receive 28% less benefit if they retired at 62 compared to if they retired at 66 years and eight months, which is the full-benefit retirement age for that cohort. Since we only know the birth year and not the birth month, we do not know their exact age (i.e., accurate at the month level). Therefore, we exclude the boundary ages (age 62 for social security eligibility and 65 for Medicare eligibility) to avoid misclassifying people. While we expect Medicaid expansion to affect 63-64-year-old respondents, there will be little or no effect among 66-67-year-old individuals since they are eligible for Medicare. Furthermore, most other social safety programs (such as worker protection or food assistance) are not age-restricted. In addition, these individuals are of similar age, so we expect their financial and health profiles to be similar. Now, any difference in state-level variation in safety programs will be differenced out.

We should note that if a family member lost a job, the HPS did not ask who that family member was or what the family member's age was. Since Medicare eligibility is based on the age of the relevant family member (not the respondent), this analysis is meaningful only when the respondent himself or herself lost a job.^6^ We show that our DDD estimates are similar to our DD estimates.

Finally, one more concern with the causal interpretation is a potential violation of the Stable Unit Treatment Value Assumption (SUTVA), which in our context may happen if individuals who are more likely to lose jobs and require mental health treatment conditional on losing jobs, systematically move to expansion states to take advantage of the Medicaid expansion. If that were the case, we would expect job-loss-induced mental distress to be worse in expansion states, which will bias our estimate downward. In other words, the estimates reported below should be considered a lower bound. However, we have reasons to believe that SUTVA is not violated here. Goodman (2017) found no increase in migration from non-expansion states to expansion states following the first round of Medicaid expansions. There is still a possibility that people may move to expansion states after losing their jobs. However, that is unlikely because we are considering job losses in the middle of a pandemic, given all the restrictions on movement during our sample period (April 2020 to March 2021).

## 4 Results

We begin with the prevalence of mental distress in expansion and non-expansion states and the corresponding mean DD estimates for all three groups. Then we estimate the effect of Medicaid expansion on job-loss-induced mental distress using a regression framework. If access to Medicaid is the reason behind the difference in outcomes across these two types of states, we should see increased Medicaid coverage among individuals in expansion states following a job loss. Therefore, next, we show that individuals in expansion states are more likely to be covered by Medicaid following a job loss than those in non-expansion states. Finally, we explore the mechanisms through which Medicaid may affect job-loss-induced mental distress.

### 4.1 Mean DD

The first two columns of Panel A of Table 3 show the prevalence of mental distress in expansion and non-expansion states in three groups. As we defined earlier, G1 consists of individuals who lost their jobs, and G2 consists of individuals who did not lose their job but a family member lost a job during the pandemic. Individuals from households without any job loss are in the third or the control group (G3). The prevalence of moderate or severe mental distress in G3 (last row of Panel A) is 0.9 percentage points higher in the expansion states (21.0%) than in the non-expansion states (20.1%). However, moderate or severe mental distress is 1.9 percentage points less prevalent in G1 in the expansion states (44.3%) than in the non-expansion states (46.2%).

**Table 3:** Prevalence of anxiety, depression, and distress.

Panel A: Mean difference-in-difference (in percentage points)				
	Exp. states mean [SD]	Non-exp. states mean [SD]	Diff. (SE)	Diff. in Diff. (SE)
Own job loss	0.443 [0.497]	0.462 [0.499]	−0.019 (0.003)***	−0.028 (0.005)***
Family job loss	0.325 [0.468]	0.329 [0.470]	−0.004 (0.002)**	−0.013 (0.004)***
No job loss	0.210 [0.408]	0.201 [0.400]	0.009 (0.001)***	–
Panel B: Mean difference-in-difference (in odds ratios)				
Own job loss	0.443 [0.497]	0.462 [0.499]	0.929 (0.010)***	0.878 (0.019)***
Family job loss	0.325 [0.468]	0.329 [0.470]	0.981 (0.009)**	0.927 (0.016)***
No job loss	0.210 [0.408]	0.201 [0.400]	1.059 (0.009)***	–

Note: ***p < 0.01, **p < 0.05, *p < 0.1. Standard deviations are in brackets and standard errors are in parenthesis.

**Table 4:** Comparing the employment and job-loss situations of 63–64-year old respondents to 66-67-year-old respondents.

	63-64-year-olds	66-67-year-olds
No COVID-19 job loss & respondent working (G3)	33.85	22.31
Own COVID-19 job loss & respondent not working (G1)	18.85	18.24
Family member COVID-19 Job loss & respondent working (G2; excluded from DDD analysis)	19.14	11.96
No COVID-19 job loss & respondent not working (excluded from DDD analysis)	28.16	47.49

Therefore, the mean DD estimate (column 4) suggests that respondents in expansion states are 2.8 percentage points (p-value<0.001) less likely to suffer from moderate or severe mental distress following own job loss (G1), and 1.3 percentage points (p-value<0.001) less likely to suffer from moderate or severe mental distress following the job loss of a family member (G2). Panel B presents the mean DD estimates using the odds ratios, which suggests that the estimated effect is about 12.2% (p-value<0.001) for G1 and about 7.3% (p-value<0.001) for G2.

### 4.2 Regression DD

Next, we estimate the regression equation (1) to control for observable differences. Before discussing the parameters of primary interest, we briefly discuss the estimates for control variables. Table A1 in the Appendix presents the odds ratios from Logit models. The first column is for moderate or severe distress, and the following two columns are for the two components: anxiety and depression. The first column shows that older respondents, female respondents are more likely to report mental distress. Married respondents, respondents with children, and non-white respondents are less likely to report mental distress. Education is not significantly associated with distress, but income is. Respondents from higher-income families are less likely to report mental distress.

**Fig. 1:**
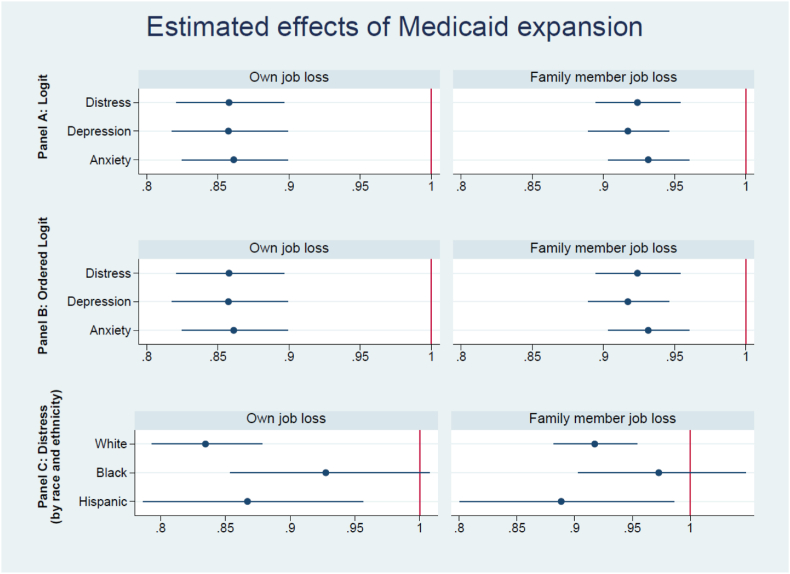
Effect of Medicaid expansion on mental distress Note1: The period covered spans from April 2020 to March 2021. Estimated odds ratios are for interaction terms from Logistic regressions. Controls include gender, marital status, age, age squared, number of children, educational categories, income categories, state-level COVID-19 cases per day, state-level COVID-19 deaths per day, survey round, and state fixed effects. Note2: 95% confidence intervals are based on standard errors clustered at the state level.

Panel A of Fig. 1 presents the results for the outcome variables: moderate or severe distress, anxiety, and depression. The left panel shows the odds ratios for G1, and the right panel presents the odds ratios for G2. The left panel shows that the odds ratio for mental distress of G1 (own job loss) is 0.858 (p-value <0.001), or in other words, respondents who live in expansion states are about 14.2% less likely to have moderate to severe mental distress after a job loss compared to those who live in non-expansion states. This estimate is similar to the mean DD estimate reported in Section 4.1. The results for the two components of mental distress (anxiety and depression) are similar to the results for overall mental distress. The odds ratio for anxiety is 0.857 (p-value<0.001) and 0.861 (p-value<0.001) for depression. These results suggest that the Medicaid expansion reduced both anxiety and depression associated with job loss.

The right panel shows that the respondents who live in expansion states are about 7.6% (p-value <0.001) less likely to have moderate to severe mental distress following the job loss of a family member (G2) than those who live in non-expansion states. The results for the two components of mental distress (anxiety and depression) are similar to the overall mental distress for G2.

We also estimated an Ordered Logit model where the outcome variable may take the values one (no mental distress; PHQ score ≤ 2), two (low mental distress; 3 ≤ PHQ score ≤ 5), three (moderate mental distress; 6 ≤ PHQ score ≤ 8), or four (severe mental distress; PHQ score ≥ 9). The estimated odds ratios from the Ordered Logit model are presented in Panel B of Fig. 1. The results are similar to the results presented in Panel A.

In Panel C, we explore the heterogeneity in the effect of Medicaid expansion across racial and ethnic groups. The estimates suggest that the protective effect of Medicaid expansion benefits all racial and ethnic groups. Medicaid expansion reduced moderate to severe distress by 16.5% (p-value <0.001) among whites, by 7.3% (p-value: 0.074) among blacks and 13.3% among Hispanics (p-value: 0.004). The relatively large effect on white respondents is somewhat surprising given that previous research has shown that Black and Hispanic Americans receive more benefits from Medicaid expansion than White Americans (Buchmueller et al., 2016; Courtemanche et al., 2017). Our result may be explained by the fact that job loss during the COVID-19 pandemic was across the board.

### 4.3 Comparability of treatment and control groups

We present the summary statistics for all the control variables in Appendix Table 2, separately for each group and by whether they live in an expansion state or not. The first two columns show the statistics for those who lost their job (G1), the next two for those who have a family member who lost a job (G2), and the final two columns are for those who did not experience any job loss during the pandemic (G3). The summary statistics show some differences across groups (for example, in expansion states, 40% of G1 has a college degree or more vs. 68% of G3). However, this is less of a concern in this study because our primary goal is not to identify how job losses affect mental health. In addition, most of the job losses were mass layoffs in nature, which means individuals lost their jobs because of economic shocks brought on by the pandemic and not because of performances or characteristics.

Since parallel trend assumption or its variants cannot be tested in the current context, we check 1) if the prevalence of COVID-19 and its impact on the economy were different in expansion and non-expansion states, or 2) if there is a significant difference-in-difference (DD) in the demographics of the individuals who lost their jobs and those who did not in expansion and non-expansion states, which may indicate an unobserved difference across groups.

In our data, there are no differences in job loss rates (p-value 0.99), SNAP participation rates (p-value 0.53), and COVID-19 death rates per million resident (p-value 0.29) among expansion and non-expansion states. It is important to note that our sample period (from April 2020 to March 2021) is mostly before vaccines became widely available in the U.S. The death rates may have differed after vaccines became widely available.

Second, there is little difference within a group across expansion and non-expansion states (for example, among G1, 40% of G1 has a college degree or more in expansion states vs. 36% in non-expansion states; column1 vs. column2). In other words, the summary statistics suggest that the type of individuals who lost jobs (G1) in expansion and non-expansion states are similar (column 1 vs. column 2). Similarly, individuals who did not have a job loss (G3) in expansion and non-expansion states are similar (column 5 vs. column 6). Therefore, the differences between the groups are also similar in expansion and non-expansion states.

To test this, we report the mean DD control variable. We create binary variables for non-binary variables (such as age, education, household income, and the number of children) by dividing the sample into two segments: below and above the median. The median respondent in the sample is 44 years old, has less than a college education, has a household income between $75,000 and $100,000, and does not have a child.

**Fig. 2:**
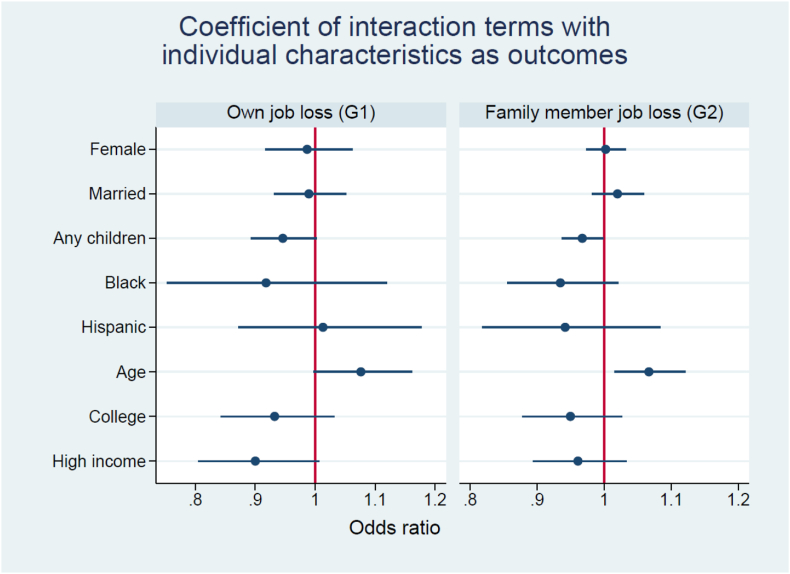
Mean Difference in Difference for controls Note1: The period covered spans from April 2020 to March 2021. For each control variable, we estimate a Logistic regression (as in eq. (1)) with group status (G1 and G2), expansion status and the corresponding interaction terms as independent variables (without any control variables). The figure above presents the estimated odds ratios corresponding to the interaction terms. Note2: 95% confidence intervals are based on standard errors clustered at the state level.

Fig. 2 shows the mean DD estimates for all the control variables. We estimate equation (1) for each control variable (as outcome) but without any additional control variables. Therefore, the coefficient of the interaction term represents the mean DD. We do not find any difference in race, ethnicity, gender, marital status, education, or income. Even though the DD estimates are statistically significant for the age variable for G1 and G2 and the proportion of respondents with children for G1, these differences are minor from a pragmatic perspective. These results suggest that the difference between respondents who lost jobs and those who did not is similar (with minor exceptions) in expansion and non-expansion states.^7^

In addition to individual characteristics, we also control for local COVID-19 conditions by using data from Johns Hopkins University (JHU) on COVID-19 activity. The JHU publishes the cumulative number of cases and deaths for each state at a daily frequency. We assign the numbers associated with the middle day of any round to that round. For example, if a round was from the first to the 15th of a month, we take the 8th day and assign the COVID-19 activity data from that day to that round of the HPS. We use these two measures of COVID-19 activity: the number of cases per day and the number of deaths per day. We normalize these numbers to the average number of cases and deaths per million residents for each state. In our data, the average number of cases per million residents is about 200, and the average number of deaths per million residents is about four.

### 4.4 Are the differences due to medicaid expansion?

**Fig. 3:**
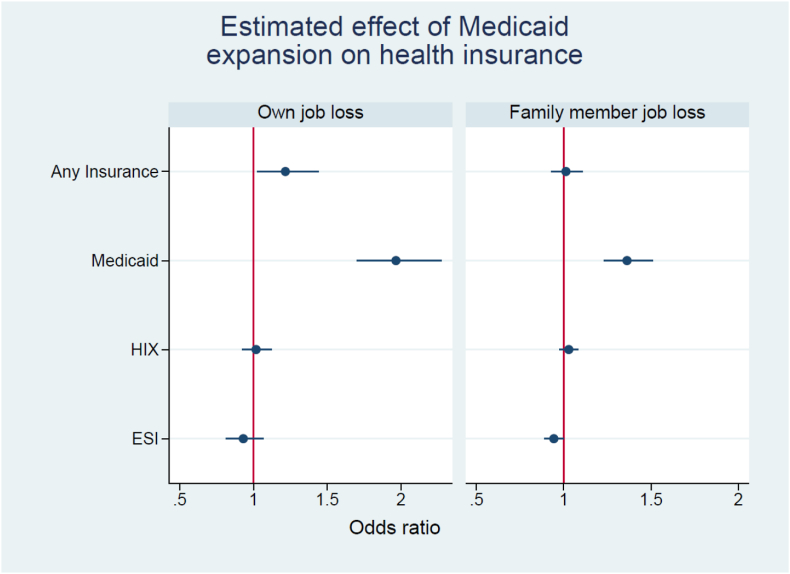
Effect of Medicaid expansion on health insurance coverage Note1: The period covered spans from April 2020 to March 2021. Estimated odds ratios are for interaction terms from Logistic regressions. Controls include gender, marital status, age, age squared, number of children, educational categories, income categories, state-level COVID-19 cases per day, state-level COVID-19 deaths per day, survey round, and state fixed effects. Note2: 95% confidence intervals are based on standard errors clustered at the state level.

If access to Medicaid is the reason behind the difference in outcomes across these two types of states, we should see increased Medicaid coverage among individuals in expansion states following a job loss. Therefore, next, we show that individuals in expansion states are more likely to be covered by Medicaid following a job loss than those in non-expansion states. We use logistic regression with the source of health insurance (Medicaid, ESI, or HIX), and whether they have health insurance from any source as the outcome variables. The estimated odds ratios for all variables (including the control variables) are shown in Appendix Table A2. The left panel of Fig. 3 shows that respondents in expansion states are 21.7% (p-value 0.024) more likely to have insurance coverage following their job loss (G1). The increased insurance coverage is driven by a 96.6% (p-value <0.001) increase in Medicaid coverage. There are no significant differences in ESI rates or insurance brought through HIX.

We do not find any effect of Medicaid expansion on overall insurance coverage following the job loss of a family member. However, our estimates show that those who live in expansion states are 36.3% (p-value <0.001) more likely to have Medicaid coupled with a 5.7% (p-value 0.054) decline in ESI coverage following a job loss of a family member than those living in non-expansion states.

### 4.5 Why? Exploring mechanisms

**Fig. 4:**
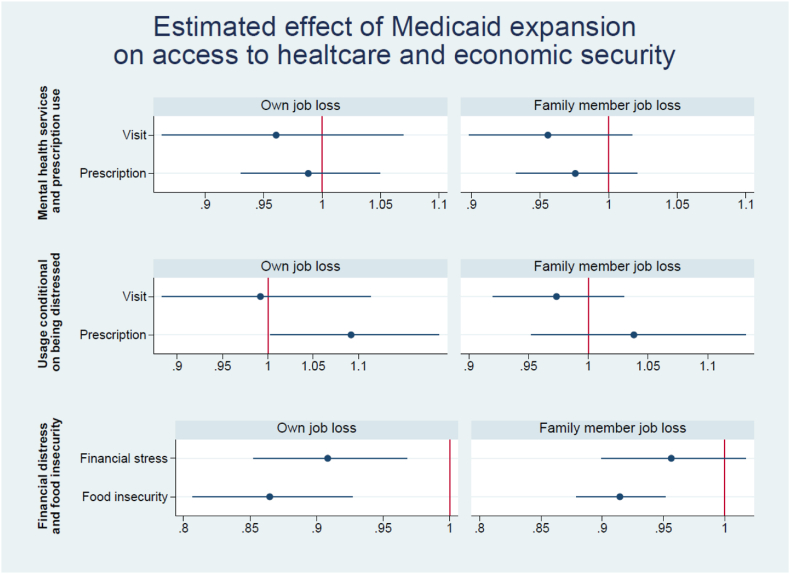
Effect of Medicaid expansion on access to healthcare and economic security Note1: The period covered spans from April 2020 to March 2021. Estimated odds ratios are for interaction terms from Logistic regressions. Controls include gender, marital status, age, age squared, number of children, educational categories, income categories, state-level COVID-19 cases per day, state-level COVID-19 deaths per day, survey round, and state fixed effects. Note2: 95% confidence intervals are based on standard errors clustered at the state level.

Next, we explore how Medicaid expansion reduces mental distress. In the Introduction, we discussed two primary mechanisms: more healthcare utilization and reduced food insecurity or financial stress. We first check whether individuals in expansion states use more mental health services than those in non-expansion states. In the HPS, respondents were asked whether they had used any mental health services (such as therapy or counseling) in the four weeks preceding an interview. They were also asked about mental-health-related prescription drug use in the two weeks preceding an interview. We use these two outcome variables to assess the effect of Medicaid expansion on access. Previous research has shown that Medicaid expansion has increased diagnosis of chronic conditions (which implies increased access and utilization of healthcare services) and prescription drug use among eligible populations, including substance abuse disorder patients (Ghosh, Simon, & Sommers, 2019; Maclean & Saloner, 2019; Miller & Wherry, 2019). The odds ratios are presented in Panel A of Fig. 4. The results suggest no difference in either mental health-related visits or prescription drug use between groups in expansion and non-expansion states. In Panel B, we present the odds ratios among those who report moderate or severe mental distress. Even in this case, mental health services usage has no effect. However, the individuals in the expansion states are 9.2% (p-value 0.043) more likely to use a prescription drug following a job loss than their counterparts in non-expansion states. We do not find any effect of Medicaid expansion on mental health care usage.

Next, we explore whether Medicaid expansion improved financial security among those who lost jobs during the pandemic. In the HPS data, the respondents were asked about general financial well-being and food security. The financial well-being question asked whether they had difficulty paying for usual household expenses (such as food, rent, and bills) in the seven days preceding an interview. The possible answers are no, little, some, or extreme difficulty. We use an Ordered Logit model to estimate the effect of Medicaid expansion on financial stress. The results are presented in Panel C of Fig. 4. The estimates suggest that individuals living in expansion states are about 9.2% (p-value 0.003) less likely to be in financial distress following a job loss than their counterparts in non-expansion states. There is no significant effect of Medicaid expansion on financial stress following the job loss of a family member.

The HPS also asked a specific question about food insecurity. As discussed earlier, food insecurity (Siefert et al., 2004; Weaver et al., 2021; Jones, 2017; Lund et al., 2010; Tribble et al., 2020) is associated with poor mental health. The estimated odds ratios from food security regression suggest that the respondents in the expansion states are about 13.5% (p-value <0.001) less likely to have moderate or severe food insecurity following a job loss than their counterparts in non-expansion states. In addition, the respondents in the expansion states are about 8.6% (p-value<0.001) less likely to have food insecurity following the job loss of a family member than their counterparts in non-expansion states. As we discussed earlier, financial stress and food insecurity can contribute to mental distress. These results suggest that Medicaid can reduce mental distress by providing more economic security.

One concern with this result may be that the expansion states also have other social safety programs. First, we should note that there are no differences in job loss rates (p-value 0.99), and SNAP participation rates (p-value 0.53) across expansion and non-expansion states. However, since we cannot check for all programs, we use a DDD structure to rule out such possibilities. We discuss that in Section 4.6.

### 4.6 Robustness checks

#### 4.6.1 Restricting the sample to respondents with high school or less education

**Fig. A1:**
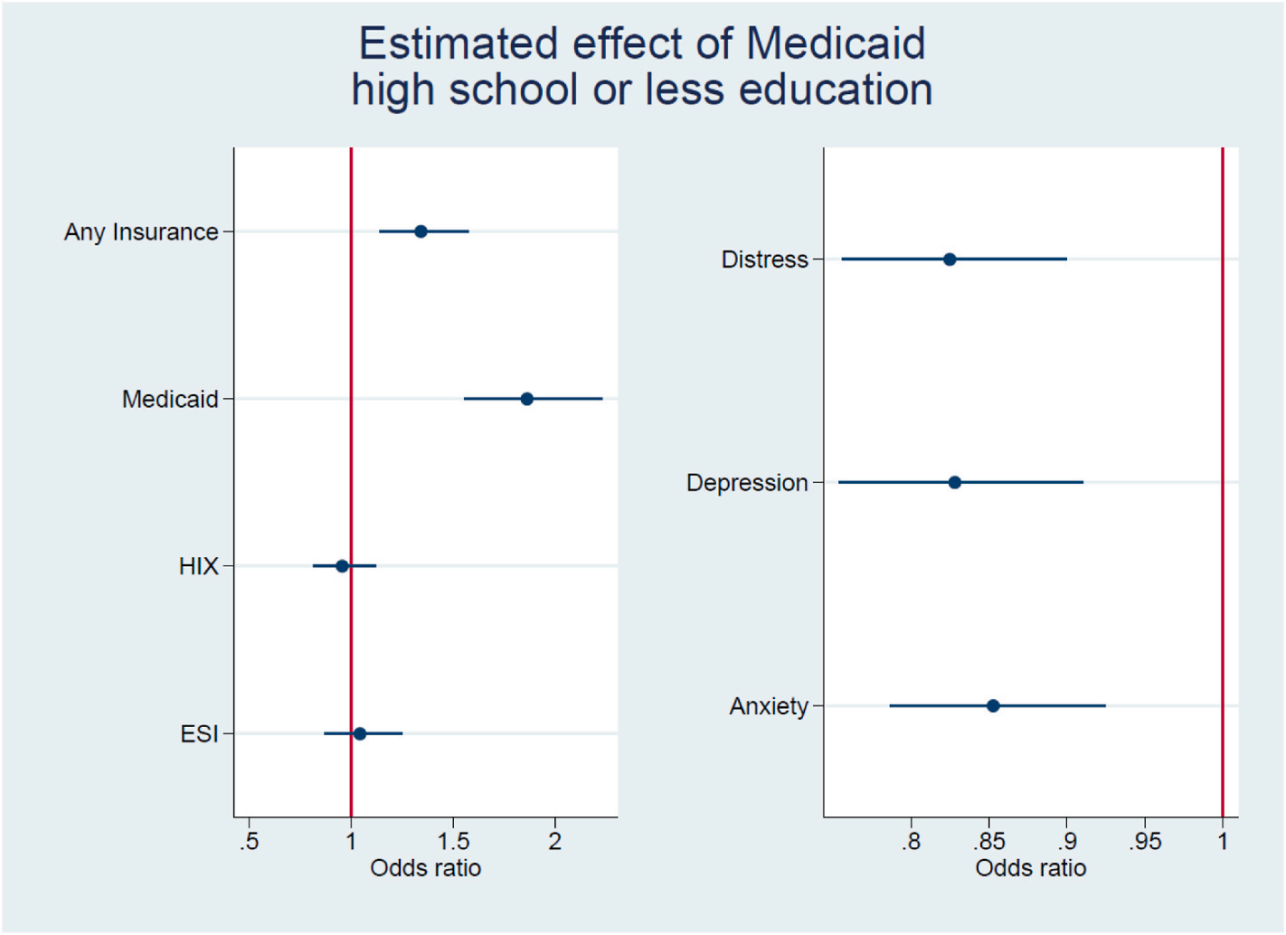
Effect of Medicaid expansion on respondents with high school or less education Note1: The period covered spans from April 2020 to March 2021. Estimated odds ratios are for interaction terms from Logistic regressions. Controls include gender, marital status, age, age squared, number of children, educational categories, income categories, state-level COVID-19 cases per day, state-level COVID-19 deaths per day, survey round, and state fixed effects. Note2: 95% confidence intervals are based on standard errors clustered at the state level.

As we discussed in the Data section, in our baseline analysis, we included all respondents. One concern with that may be the potential for increased unobserved heterogeneity. Therefore, in this section, we restrict our sample to respondents with high school or less education to evaluate the effect of Medicaid expansion. For this part, we focus only on G1 (own job loss). The estimated odds ratios, presented in Figure A1 in the Appendix, show that the Medicaid expansion increased Medicaid coverage by 40.1% and correspondingly reduced moderate to severe mental distress by 17.5% in the expansion states following a job loss.

#### 4.6.2 Using medicare eligibility as a placebo test

To ensure that our results are not driven by unobserved state-level differences (such as social safety programs that may asymmetrically affect the employed and unemployed), we compare the effect of Medicaid expansion on respondents just below 65 (63–64) and above 65 (66–67). While we expect Medicaid expansion to affect 63–64-year-old respondents, there will be little or no effect among 66-67-year-old individuals since they are eligible for Medicare. The advantage of DDD is that even in the presence of differences in social safety programs across expansion and non-expansion states, the DDD estimates will still be consistent, as long as those programs are also not age-restricted.

Notably, a substantial number (7.2 million^8^) of low-income individuals above 65 are eligible for both Medicaid and Medicare (dual eligible). While there is substantial inter-state variation in the dual-eligibility criteria (Miller, Hu, Kaestner, Mazumder, & Wong, 2021), most states restrict dual eligibility to low-income, low-asset, and disabled seniors (such as seniors on Supplemental Security Income or SSI, which only allows assets up to $2000 for individuals and $3000 for couples). Therefore, we expect the impact of Medicaid expansion to be limited to none among people above 65.

**Fig. 5:**
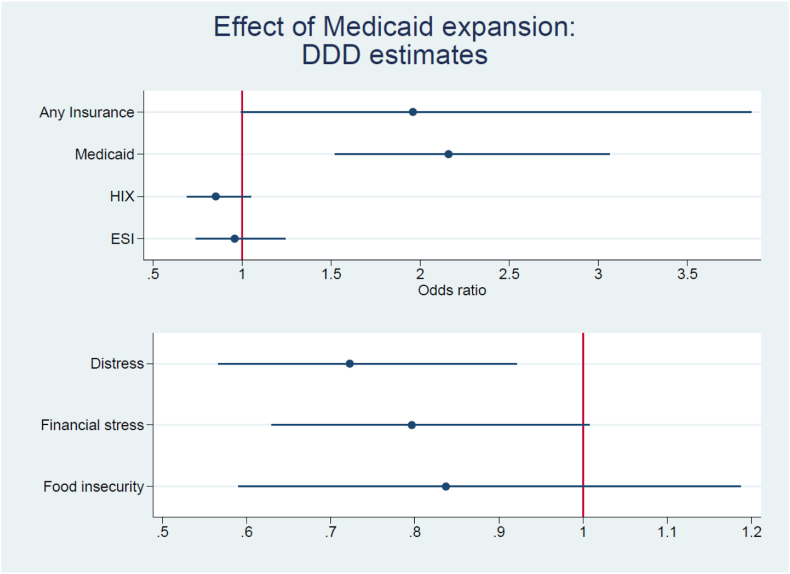
DDD estimates from 63 to 67 year old respondents Note1: The period covered spans from April 2020 to March 2021. Estimated odds ratios are for interaction terms from Logistic regressions. Controls include gender, marital status, age, age squared, number of children, educational categories, income categories, state-level COVID-19 cases per day, state-level COVID-19 deaths per day, survey round, and state fixed effects. Note2: 95% confidence intervals are based on standard errors clustered at the state level.

Fig. 5 presents the results. As we argue in Section 3.2, this analysis is meaningful only when the respondent lost a job. The odds ratios from the DDD regression on insurance status (top panel) show that the Medicaid expansion increased the probability of Medicaid coverage by 115% among those who lost their jobs in expansion states. However, there is no statistically significant change in coverage through employers (ESI) or the marketplace (HIX). Consequently, Medicaid expansion reduced moderate to severe mental distress by 28% (bottom panel).^9^ Again consistent with previous results, we find that Medicaid expansion reduces mental distress through reduced food insecurity. Given that the DDD estimates are similar to DD results, we conclude that our estimated effect is indeed the effect of Medicaid expansion.

## 5 Conclusion

One of the goals of social safety programs is to work as a shock absorber in times of crisis. The COVID-19 pandemic was a once-in-a-century health crisis. We show that the Medicaid expansion mitigated the adverse effects of job loss on mental health during the COVID-19 pandemic. Moreover, we show that the economic security provided by Medicaid is as important (if not more) as access to healthcare. While the access to healthcare aspect of health insurance in general and Medicaid, in particular, has been widely discussed, the economic security aspect of Medicaid has received less attention. This paper shows that Medicaid worked as a significant shock absorber in a time of great health crisis, not just by providing more access to healthcare but also by providing economic security during the COVID-19 pandemic.

These results are relevant as governments around the world are looking for policies to protect vulnerable populations from ever-increasing food insecurity. It is estimated that more than 800 million people^10^ are food insecure, and more than 300 million people^11^ are facing severe food insecurity. This includes more than 38 million Americans. Expansion of social safety nets that reduce food insecurity may provide not only reduce hunger but they may provide significant benefits to the mental health of these vulnerable populations.

## Funding

None.

## Ethics statement

I declare no conflict of interest. I am solely responsible for all the contents of this paper.

## Author statements

As the sole author, Sankar Mukhopadhyay is solely responsible for all the contents of this paper.

## Declaration of competing interest

None.

## Figures and Tables

**Table A1:** Estimated odds ratios for Mental health outcomes

VARIABLES	(1)	(2)	(3)
	Distress	Anxiety	Depression
Age	1.023***	1.015***	1.023***
	(0.00209)	(0.00197)	(0.00216)
Age squared	1.000***	1.000***	1.000***
	(2.57e-05)	(2.40e-05)	(2.61e-05)
Female	1.333***	1.117***	1.461***
	(0.00919)	(0.00863)	(0.0106)
Married	0.801***	0.756***	0.851***
	(0.00622)	(0.00594)	(0.00596)
Black	0.792***	0.872***	0.757***
	(0.0127)	(0.0136)	(0.0135)
Asian	0.764***	0.882***	0.664***
	(0.0127)	(0.0159)	(0.0107)
Other	1.091***	1.118***	1.070***
	(0.0138)	(0.0136)	(0.0159)
Hispanic	0.919***	0.936***	0.917***
	(0.0199)	(0.0175)	(0.0175)
Education (less than H.S. is base)			
Some HS	0.994	0.998	1.019
	(0.0383)	(0.0338)	(0.0455)
HS degree	0.952	0.945	0.984
	(0.0460)	(0.0427)	(0.0514)
Some college	1.061	1.020	1.122**
	(0.0544)	(0.0485)	(0.0638)
Assoc. degree	0.967	0.922	1.040
	(0.0526)	(0.0467)	(0.0615)
College deg.	0.930	0.826***	1.055
	(0.0518)	(0.0430)	(0.0646)
Graduate deg.	0.942	0.791***	1.125**
	(0.0486)	(0.0381)	(0.0630)
Income (<25K is base)			
$25K - $35K	0.895***	0.898***	0.900***
	(0.0103)	(0.0105)	(0.0108)
$35K - $50K	0.840***	0.833***	0.846***
	(0.0122)	(0.0121)	(0.0134)
$50K - $75K	0.748***	0.733***	0.764***
	(0.0126)	(0.0123)	(0.0137)
$75K - $100K	0.642***	0.622***	0.673***
	(0.0127)	(0.0115)	(0.0132)
$100K - $150K	0.557***	0.533***	0.600***
	(0.0140)	(0.0122)	(0.0141)
$150K - $200K	0.498***	0.466***	0.546***
	(0.0134)	(0.0123)	(0.0144)
>$200,000K	0.428***	0.387***	0.480***
	(0.0118)	(0.0105)	(0.0132)
# of children (0 is base)			
One	0.926***	0.886***	0.980***
	(0.00733)	(0.00897)	(0.00776)
Two	0.869***	0.804***	0.955***
	(0.00861)	(0.00824)	(0.00937)
Three	0.822***	0.762***	0.894***
	(0.0126)	(0.0109)	(0.0142)
Four	0.789***	0.758***	0.844***
	(0.0224)	(0.0201)	(0.0215)
Five	0.788***	0.760***	0.834***
	(0.0282)	(0.0285)	(0.0267)
Daily # of cases	1.000***	1.000***	1.000***
	(4.23e-05)	(4.31e-05)	(4.33e-05)
Daily # of deaths	1.003	1.004***	1.002
	(0.00187)	(0.00155)	(0.00189)
Own job loss (G1)	2.852***	2.816***	2.718***
	(0.0440)	(0.0476)	(0.0358)
Family member job loss (G2)	1.806***	1.755***	1.804***
	(0.0215)	(0.0194)	(0.0190)
Expansion*G1	0.858***	0.857***	0.861***
	(0.0193)	(0.0208)	(0.0190)
Expansion*G2	0.924***	0.917***	0.931***
	(0.0151)	(0.0145)	(0.0145)
Constant	0.350***	0.439***	0.367***
	(0.0269)	(0.0309)	(0.0334)

Observations	872,428	872,428	872,428

Note: Estimated odds ratios, standard errors in parentheses; clustered at the state level. Variables included but not shown, state dummies and round dummies.

***p < 0.01, **p < 0.05, *p < 0.1.

**Table A2:** Estimated odds ratios for health insurance coverage

VARIABLES	(1)	(2)	(3)	(4)
	Any insurance	Medicaid	HIX	ESI
Age	0.924***	1.077***	0.942***	0.974***
	(0.00429)	(0.00734)	(0.00301)	(0.00378)
Age squared	1.001***	0.999***	1.001***	1.000***
	(5.23e-05)	(7.82e-05)	(3.44e-05)	(4.32e-05)
Female	1.504***	1.481***	0.943***	1.195***
	(0.0252)	(0.0287)	(0.00906)	(0.0125)
Married	1.461***	0.757***	1.084***	1.436***
	(0.0243)	(0.0153)	(0.0143)	(0.0250)
Black	1.109***	1.334***	1.269***	1.370***
	(0.0330)	(0.0377)	(0.0274)	(0.0443)
Asian	0.979	1.220***	1.615***	1.202***
	(0.0452)	(0.0491)	(0.0461)	(0.0401)
Other	1.102	1.188***	1.055**	0.939*
	(0.0741)	(0.0231)	(0.0221)	(0.0323)
Hispanic	0.759***	0.958*	1.122***	1.037
	(0.0347)	(0.0225)	(0.0395)	(0.0248)
Education (less than H.S. is base)				
Some HS	1.283***	1.268***	0.987	1.085
	(0.0485)	(0.0648)	(0.0432)	(0.0571)
HS degree	1.595***	1.195***	1.052	1.488***
	(0.0626)	(0.0702)	(0.0470)	(0.0762)
Some college	2.044***	1.185***	1.014	1.563***
	(0.0819)	(0.0697)	(0.0450)	(0.0811)
Assoc. degree	2.297***	1.084	1.030	1.730***
	(0.0902)	(0.0666)	(0.0484)	(0.0903)
College deg.	3.171***	0.841***	0.982	1.941***
	(0.133)	(0.0552)	(0.0495)	(0.119)
Graduate deg.	4.034***	0.709***	0.899*	2.075***
	(0.156)	(0.0433)	(0.0494)	(0.128)
Income (<25K is base)				
$25K - $35K	0.939	0.462***	1.404***	1.919***
	(0.0408)	(0.0153)	(0.0361)	(0.0507)
$35K - $50K	1.108*	0.259***	1.433***	3.179***
	(0.0604)	(0.0107)	(0.0585)	(0.102)
$50K - $75K	1.514***	0.134***	1.345***	5.370***
	(0.0977)	(0.00703)	(0.0623)	(0.182)
$75K - $100K	2.191***	0.0697***	1.202***	8.325***
	(0.152)	(0.00396)	(0.0607)	(0.332)
$100K - $150K	3.497***	0.0387***	1.063	12.47***
	(0.233)	(0.00250)	(0.0535)	(0.504)
$150K - $200K	4.979***	0.0259***	1.015	14.97***
	(0.322)	(0.00181)	(0.0478)	(0.607)
>$200,000K	7.213***	0.0165***	1.030	15.07***
	(0.574)	(0.00141)	(0.0454)	(0.846)
# of children (0 is base)				
One	1.276***	2.208***	1.052***	0.990
	(0.0272)	(0.0975)	(0.0104)	(0.00854)
Two	1.446***	3.164***	1.054***	0.920***
	(0.0481)	(0.149)	(0.0122)	(0.0125)
Three	1.424***	4.464***	1.062***	0.768***
	(0.0453)	(0.232)	(0.0174)	(0.0148)
Four	1.491***	6.215***	1.060*	0.636***
	(0.0773)	(0.398)	(0.0359)	(0.0224)
Five	1.300***	8.860***	0.972	0.490***
	(0.0685)	(0.683)	(0.0422)	(0.0180)
Daily # of cases	1.000	1.000	1.000	1.000**
	(5.24e-05)	(4.90e-05)	(2.57e-05)	(3.58e-05)
Daily # of deaths	1.004*	1.002	1.002**	1.000
	(0.00230)	(0.00228)	(0.000970)	(0.00132)
Own job loss (G1)	0.181***	1.797***	1.265***	0.165***
	(0.0130)	(0.121)	(0.0445)	(0.0106)
Family member job loss (G2)	0.420***	1.358***	1.233***	0.475***
	(0.0141)	(0.0658)	(0.0237)	(0.0114)
Expansion*G1	1.217**	1.966***	1.018	0.931
	(0.106)	(0.146)	(0.0513)	(0.0644)
Expansion*G2	1.013	1.363***	1.029	0.943*
	(0.0459)	(0.0712)	(0.0275)	(0.0285)
Constant	15.45***	0.0204***	0.398***	1.285**
	(2.094)	(0.00277)	(0.0395)	(0.137)

Observations	872,428	872,428	872,428	872,428

Note: Estimated odds ratios, standard errors in parentheses; clustered at the state level. Variables included but not shown, state dummies and round dummies.

***p < 0.01, **p < 0.05, *p < 0.1.

**Table A3:** DDD regression parameter estimates

	Mental distress
Lost job	2.191***
	(0.132)
Expansion* Lost job	1.023
	(0.084)
63-64-year-old	1.052
	(0.103)
Expansion*63-64-year-old	1.036
	(0.083)
63-64-year-old*Lost job	1.314***
	(0.118)
Expansion*63-64-year-old* Lost job	0.722***
	(0.089)
Sample size	40,970

Note: Estimated odds ratios, standard errors in parentheses; clustered at the state level. Controls include gender, marital status, age, age squared, number of children, educational categories, income categories, state-level COVID-19 cases per day, state-level COVID-19 deaths per day, survey round, and state fixed effects.

***p < 0.01, **p < 0.05, *p < 0.1.

## Data Availability

Data will be made available on request.

## Footnotes

1 Unemployment Insurance Weekly Claims, Department of Labor, May 14, 2020. https://www.dol.gov/ui/data.pdf.

2 https://www.bls.gov/news.release/pdf/empsit.pdf.

3 https://www.pewresearch.org/fact-tank/2021/02/10/unemployed-americans-are-feeling-the-emotional-strain-of-job-loss-most-have-considered-changing-occupations/.

4 The ACA mandated Medicaid expansion for all states. A Supreme Court ruling made the expansion optional for states. A total of 37 states (and Washington DC) expanded Medicaid before 1/1/2020. Nebraska started enrolling residents under the Medicaid expansion on 08/01/2020. We classify Nebraska as an expansion state. The results of this paper remain unchanged if we drop Nebraska.

5 This rule was changed in the American Rescue Plan Act (ARPA) in March of 2021. However, our data does not include the period after ARPA.

6 Table 4 compares the employment and job-loss situations of 62–63-year old respondents to 66-67-year-old respondents. In the HPS data, these two groups report similar rates of COVID-19-induced job loss.

7 The coefficient estimate for G1 for income is significant at 10%. Since lower income is associated with more mental distress, this suggests that if anything, we are underestimating the effects of Medicaid and our estimates are lower bounds of the true effects.

8 https://www.medicaid.gov/medicaid/eligibility/seniors-medicare-and-medicaid-enrollees/index.html#:∼:text=In%20total%2C%2012%20million%20people,both%20optional%20and%20mandatory%20categories., Accessed on 05/09/2021.

9 Table A3 shows the parameter estimates from the DDD regression. The estimates suggest that the baseline mental distress is similar in these age groups. However, a job loss has a significantly more (31.4%) adverse impact on 63-64-year-olds compared to 66-67-year-olds in the non-expansion states. This may be due to health insurance as well as differences in social security benefits. As we discussed even though both groups are eligible for social security benefits, 63-64-year-olds are not eligible for full benefits. However, for respondents living in the expansion states, the effect of job loss is not significantly different between the two age groups. The reduced social security payments for the younger group apply in both expansion and non-expansion states but the Medicaid is more accessible only in the expansion states. Therefore, these results suggest that access to health insurance is the primary driver of differences in mental distress in these two age groups.

10 https://www.worldbank.org/en/topic/agriculture/brief/food-security-update.

11 https://reliefweb.int/report/world/g7-statement-global-food-security-elmau-28-june-2022.

## References

[bib1] Abbott A. (2021). COVID's mental-health toll: How scientists are tracking a surge in depression. Nature.

[bib2] Agarwal S.D., Sommers B.D. (2020). Insurance coverage after job loss-the importance of the ACA during the covid-associated recession. New England Journal of Medicine.

[bib3] Baicker K., Taubman S.L., Allen H.L., Bernstein M., Gruber J.H., Newhouse J.P., Finkelstein A.N. (2013). The Oregon experiment-effects of Medicaid on clinical outcomes. New England Journal of Medicine.

[bib4] Brooks T., Roygardner L., Artiga S., Pham O., Dolan R. (2019).

[bib5] Buchmueller T.C., Levinson Z.M., Levy H.G., Wolfe B.L. (2016). Effect of the Affordable Care Act on racial and ethnic disparities in health insurance coverage. American Journal of Public Health.

[bib6] Butterworth P., Rodgers B., Windsor T.D. (2009). Financial hardship, socioeconomic position and depression: Results from the PATH through life survey. Social Science & Medicine.

[bib7] Charles K.K., Stephens M. (2004). Disability, job displacement and divorce. Journal of Labor Economics.

[bib8] Clark A., Georgellis Y., Sanfey P. (2001). Scarring: The psychological impact of past unemployment. Economica.

[bib9] Courtemanche C., Marton J., Ukert B., Yelowitz A., Zapata D. (2017). Early impacts of the Affordable Care Act on health insurance coverage in Medicaid expansion and non-expansion states. Journal of Policy Analysis and Management.

[bib10] Cygan‐Rehm K., Kuehnle D., Oberfichtner M. (2017). Bounding the causal effect of unemployment on mental health: Nonparametric evidence from four countries. Health Economics.

[bib11] Dias F.A., Chance J., Buchanan A. (2020). The motherhood penalty and the fatherhood premium in employment during covid-19: Evidence from the United States. Research in Social Stratification and Mobility.

[bib12] Doty M.M., Collins S.R., Rustgi S.D., Kriss J.L. (2008). Seeing red: The growing burden of medical bills and debt faced by U.S. families. Issue Brief (Public Policy Institute, American Association of Retired Persons).

[bib13] Elgar F.J., Pickett W., Pförtner T.-K., Gari'epy, Genevi'eve, Gordon D., Georgiades K., Davison C. (2021). Relative food insecurity, mental health and wellbeing in 160 countries. Social Science & Medicine.

[bib14] Fronstin P., Woodbury S.A. (2020). https://research.upjohn.org/externalpapers/90/.

[bib15] Ghosh A., Simon K., Sommers B.D. (2019). The effect of health insurance on prescription drug use among low-income adults: Evidence from recent medicaid expansions. Journal of Health Economics.

[bib16] Golberstein E., Gonzales G., Sommers B.D. (2015). California's early ACA expansion increased coverage and reduced out-of-pocket spending for the state's low-income population. Health Affairs.

[bib17] Goodman L. (2017). The effect of the affordable care act medicaid expansion on migration. Journal of Policy Analysis and Management.

[bib18] Hernández R. (2020).

[bib19] Himmelstein G. (2019). Effect of the affordable care act's medicaid expansions on food security, 2010-2016. American Journal of Public Health.

[bib20] Hu L., Kaestner R., Mazumder B., Miller S., Wong A. (2016).

[bib21] Jones A.D. (2017). Food insecurity and mental health status: A global analysis of 149 countries. American Journal of Preventive Medicine.

[bib22] Lund C., Breen A., Flisher A.J., Kakuma R., Corrigall J., Joska J.A., Patel V. (2010). Poverty and common mental disorders in low and middle income countries: A systematic review. Social Science & Medicine.

[bib23] Maclean J.C., Saloner B. (2019). The effect of public insurance expansions on substance use disorder treatment: Evidence from the affordable care act. Journal of Policy Analysis and Management.

[bib24] Miller S., Hu L., Kaestner R., Mazumder B., Wong A. (2021). The ACA Medicaid expansion in Michigan and financial health. Journal of Policy Analysis and Management.

[bib25] Miller S., Wherry L.R. (2019). Four years later: Insurance coverage and access to care continue to diverge between ACA medicaid expansion and non-expansion states. AEA Papers and Proceedings.

[bib27] Schaller J., Stevens A.H. (2015). Short-run effects of job loss on health conditions, health insurance, and health care utilization. Journal of Health Economics.

[bib28] Siefert K., Heflin C.M., Corcoran M.E., Williams D.R. (2004). Food insufficiency and physical and mental health in a longitudinal survey of welfare recipients. Journal of Health and Social Behavior.

[bib29] Sorsdahl K., Slopen N., Siefert K., Seedat S., Stein D.J., Williams D.R. (2011). Household food insufficiency and mental health in South Africa. Journal of Epidemiology & Community Health.

[bib30] Sullivan D., Von Wachter T. (2009). Job displacement and mortality: An analysis using administrative data. Quarterly Journal of Economics.

[bib31] Tribble A.G., Maxfield A., Hadley C., Goodman M. (January 15, 2020). https://ssrn.com/abstract=3520061.

[bib32] Weaver L.J., Owens C., Tessema F., Kebede A., Hadley C. (2021).

